# Reduction of Torsion‐Detorsion‐Induced Testicular Damage With Hawthorn Extract: Oxidative, Hormonal, and Histological Effects

**DOI:** 10.1002/fsn3.70211

**Published:** 2025-05-13

**Authors:** Ümmü Gülşen Bozok, Gülbahar Böyük Özcan, Fatma Uysal Cinar

**Affiliations:** ^1^ Department of Physiology, Faculty of Medicine Ankara Medipol University Ankara Türkiye; ^2^ Department of Histology and Embryology Faculty of Medicine Ankara Medipol University Ankara Türkiye

**Keywords:** hawthorn extract, histopathology, oxidative stress, testicular torsion, testosterone

## Abstract

This study aimed to find out how hawthorn extract protects against damage caused by torsion/detorsion (T/D). In the study, four groups were formed, each consisting of eight rats: control, T/D, low‐dose hawthorn extract (T/D + LD), and high‐dose hawthorn extract (T/D + HD). The effect of testosterone hormone and oxidative stress parameters of total antioxidant level (TAS), total oxidant level (TOS), as well as oxidative stress index (OSI) were evaluated. We examined the histopathological effects of the hawthorn extract. Additionally, sperm count and motility were analyzed. The analyses were conducted using the IBM SPSS v22 program, and a *p*‐value of less than 0.05 was deemed statistically significant. Testosterone, TAS, TOS, and OSI parameters show statistical differences between the groups (*p* = 0.014, *p* = 0.009, *p* = 0.021, *p* = 0.004, respectively). Group differences in testicular volume are statistically significant (*p* = 0.001). The sperm count exhibits a statistically significant difference between the groups (*p* = 0.00). Sperm motility was affected only by groups at a statistically significant level (*p* = 0.00). Histopathologically, tissue damage decreased in the hawthorn extract groups compared to the T/D groups. Hawthorn extract has the potential to alleviate T/D‐induced damage by reducing oxidative stress and protecting testicular tissue. The findings suggest that hawthorn extract may have therapeutic potential in mitigating oxidative damage caused by testicular torsion and providing sustained protective effects. Longer‐term studies should explore the effects of hawthorn extract in greater detail, as these findings indicate that it may be a promising treatment option for acute testicular injury.

## Graphical abstract

Hawthorn extract reduces oxidative stress and prevents testicular damage in a torsion–detorsion model. Low‐dose and high‐dose hawthorn extract preserve testicular histology by enhancing spermatogenesis, as evidenced by an increase in late spermatid counts. Testosterone levels increased compared to both the control and torsion‐detorsion groups, suggesting that this finding could serve as a basis for further research on male infertility.

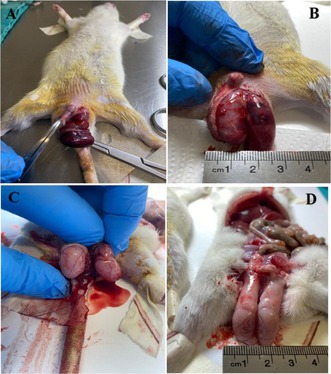

## 1 Introduction

The most common surgical urogenital emergency is testicular torsion (TT) in children, while it is considered the second most common surgical emergency in young males. Estimates show that one in every four thousand men over the age of 25 is affected by testicular torsion (Jacobsen et al. 2020). The extent of damage depends on the duration and severity of ischemia. Reperfusion restores blood flow to the testicular tissue; however, during this process, the reoxygenation of the tissue leads to the production of reactive oxygen species (ROS) (Bozok et al. 2022). T/D is an ischemia/reperfusion (I/R) injury (Küçük et al. 2021). Long‐term damage within testicular tissue is observed following detorsion. Notably, studies have shown that intense oxidative activity persists even 7 days after detorsion, leading to damage in both the ischemic and contralateral testis. In TT, preserving the testis is crucial. The most effective treatment method for TT is surgical intervention, which includes detorsion and testicular fixation or orchiectomy, depending on the extent of testicular damage (Capraro et al. 2008). Testicular atrophy can happen after detorsion because ROS production and the antioxidant defense system are out of balance. Furthermore, excessive ROS production in the testis causes direct damage to testicular tissue, leading to a decrease in sperm count and viability, lipid imbalance, and increased DNA damage, all of which ultimately contribute to male infertility. Therefore, reducing oxidative stress in testicular tissue following detorsion is crucial. Recently, antioxidants have been the focus of promising studies aimed at preventing testicular atrophy and damage (Abadi et al. 2023; Ekşi et al. 2020; Moradi‐Ozarlou et al. 2020; Yuluğ et al. 2014). Flavonoids are one of the key antioxidant compounds. They are characterized by their polyphenolic structures and exhibit potent antioxidant and anti‐inflammatory properties (Maleki et al. 2019). With these properties, flavonoids contribute to the protection of testicular tissue by reducing oxidative stress and improving sperm quality. Additionally, they exert their gonadoprotective effects through the STAT and NF‐κB pathways, further enhancing their protective role in testicular health (Abadi et al. 2023; Seker et al. 2024). One of these flavonoids is the hawthorn (*Crataegi fructus*), also known as thorn, maybush, or whitehorn (Cui et al. 2006). In vitro investigations have demonstrated its anti‐apoptotic effects through the elimination of ROS in keratinocytes (Liu et al. 2019) and its anti‐tumorigenic characteristics in B‐cell lymphoma and melanoma (Liu et al. 2019; Mustapha et al. 2016). Research on macrophages indicates that it diminishes foam cell formation through its anti‐inflammatory effects (Bai et al. 2024) and possesses immunomodulatory capabilities (Liao et al. 2024). In neuronal cells, it is proposed to enhance anti‐aging mechanisms through its antioxidant properties (Wang et al. 2022). In vivo studies have demonstrated the anti‐inflammatory (Seyidoglu et al. 2024), antioxidant (Liu et al. 2019), anti‐apoptotic (Wang et al. 2019), anti‐tumoral (Mustapha et al. 2016), anti‐lipidemic (Mao et al. 2022), anti‐glycemic (Gu et al. 2023), anti‐neurodegenerative (Zhang et al. 2022), and anti‐atherosclerotic (Koch and Malek 2011) properties of hawthorn extract. It has been found to serve as a strong immunomodulator by augmenting the overall quantity and proportion of T and B splenocytes (Lis et al. 2020). One of the primary mechanisms by which hawthorn extract exerts its biological effects may be the prevention of lipid peroxidation through the inhibition of ROS generation (Wang et al. 2011). Accordingly, we hypothesized that hawthorn extract could protect the structure and function of testicular tissue by reducing long‐term oxidative stress induced by TT.

The aim of this study is to evaluate the protective effects of hawthorn extract in a T/D model. We aimed to investigate the potential therapeutic role of hawthorn extract in mitigating oxidative stress, hormonal changes, and histological damage caused by testicular torsion. Additionally, we sought to determine the effects of different doses of hawthorn extract on testicular tissue and function, assessing whether it could serve as an effective agent in reducing tissue damage following testicular torsion. We selected hawthorn extract for our study due to its well‐documented minimal toxicity and side effects (Kao et al. 2005), its accessibility, cost‐effectiveness, and strong antioxidant properties as a natural product. Consequently, we demonstrated the effects of hawthorn extract at different doses on testicular tissue and function in animals subjected to a testicular torsion model.

Our findings suggest that hawthorn extract may offer protective benefits by mitigating some of the adverse effects associated with testicular torsion. Future studies should aim to elucidate the underlying mechanisms of these protective effects and investigate their potential applications in humans, ultimately enhancing access to alternative therapeutic options.

## 2 Materials and Methods

### 2.1 Study Groups

The sample size required for the study was determined based on the effect size for the One‐Way Analysis of Variance (ANOVA) test, calculated as *η* ^2^ = 0.30 (*f* = 0.67). This estimation ensured a statistical power of 80% and a confidence level of 95%. Consequently, a total of 32 rats were included in the study, with 8 rats allocated to each group. The study's sample size was determined utilizing the Gpower 3.1 software. The Wistar albino male animals to be used in the study were obtained from 32 Nesa Experimental Animals Laboratory R&D and Consultancy Industry and Trade Ltd. Co. at their expense. The animals were arbitrarily allocated into four groups. Group 1 was the control (C), Group 2 was T/D, Group 3 was 100 mg/kg hawthorn extract + T/D (LD + T/D), and Group 4 was 200 mg/kg hawthorn extract + T/D (HD + T/D), with 8 animals in each group. Anesthesia caused one animal to die during the study, leading to the transfer of one animal from the control group to the experimental group, leaving the control group with seven animals.

### 2.2 Development of the Experimental Model

**FIGURE 1:**
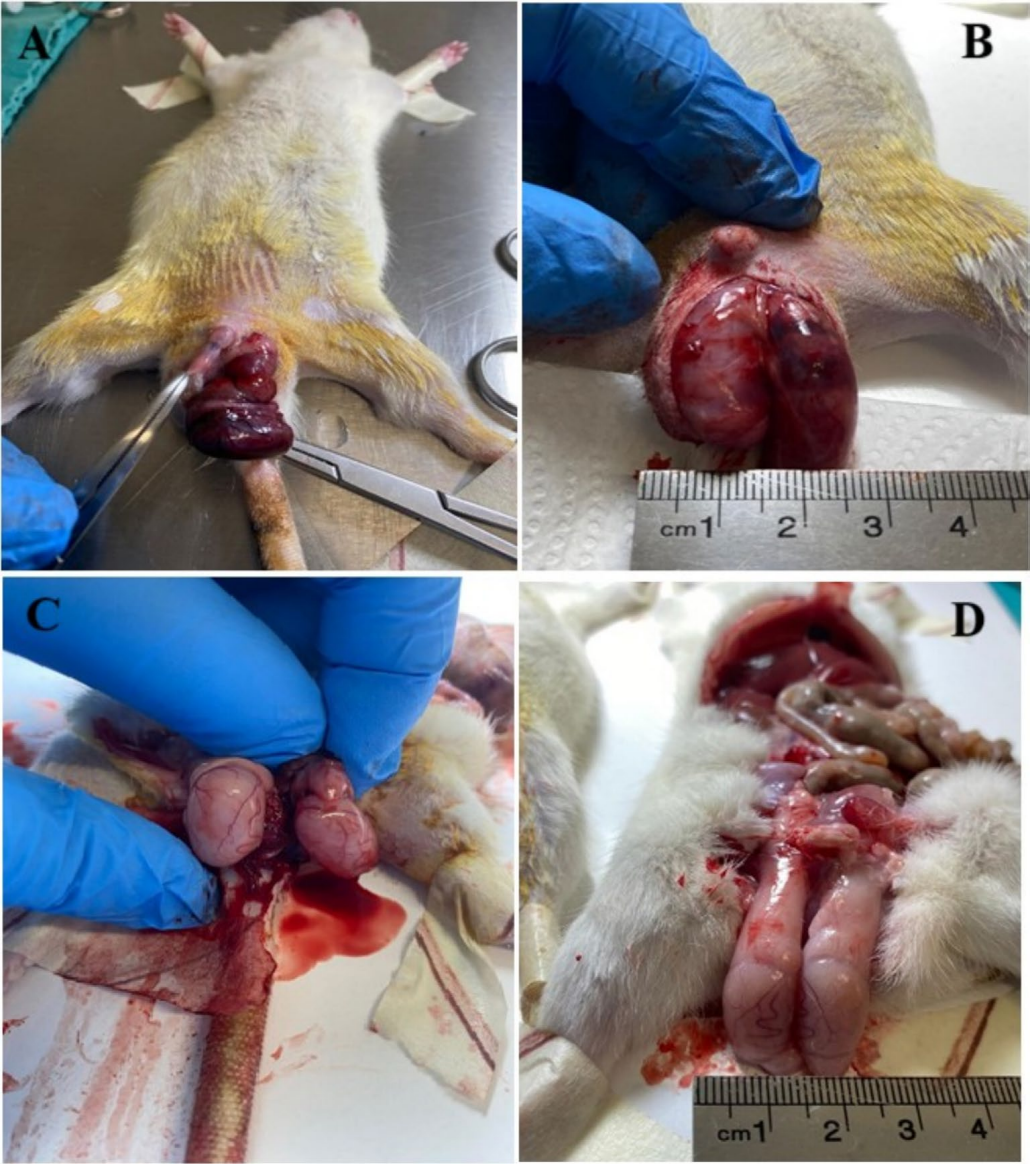
Left testicular torsion and detorsion steps. (A, B) Torsion of the left testis; (C, D) Detortion of the left testis. After performing a left inguinoscrotal incision, 720° clockwise rotation was performed on the left testicle (A, B), then fixed in the hemiscrotum with atraumatic silk sutures. Following 120 min of ischemia, the left testicle was detorsioned (C, D). Sodium heparin (500 IU/kg) was administered via the tail's peripheral vein.

All surgical groups received intraperitoneal (ip) management of ketamine (35–50 mg/kg) and xylazine (5–10 mg/kg) for anesthetic purposes. Group 1 underwent only a midline laparotomy. Following a left inguinoscrotal incision, the Group 2 T/D group induced unilateral testicular torsion (TT) by rotating the left testicle 720° in a clockwise direction (Figure 1A,B). The testicle was then secured within the hemiscrotum using atraumatic silk sutures. After 120 min of ischemia, the rats underwent a spermatic cord detorsion procedure, followed by 120 min of reperfusion (Yesil et al. 2022), (Figure 1C,D). To maintain reperfusion after ischemia, sodium heparin (500 IU/kg) was infused through the peripheral vein of the tail (Yuluğ et al. 2014).

For Groups 3 and 4, 100 mg/kg hawthorn extract and 200 mg/kg hawthorn extract (Te‐Ha Agricultural Consultancy Industry Trade.‐015071‐03.11.2022) were purchased and administered intraperitoneally to the T/D, 30 min before the ischemic procedure (Hosseinimehr et al. 2007). Subsequently, it was concluded with the application of the T/D model and heparin as described in Group 2.

Euthanasia was performed by collecting blood from the abdominal aorta under ketamine (35–50 mg/kg) and xylazine (5–10 mg/kg) anesthesia. Prior to sacrifice, the animals were evaluated for the absence of reflexes, including the toe pinch reflex and corneal reflex, to confirm unconsciousness. Only after ensuring complete unconsciousness was the final euthanasia procedure carried out. After the cessation of heart rate and respiration, testicular tissue was collected for histopathological analysis. Additionally, seminal vesicle samples were collected for sperm count and motility assessment.

### 2.3 Sperm Count and Motility Analysis

**FIGURE 2:**
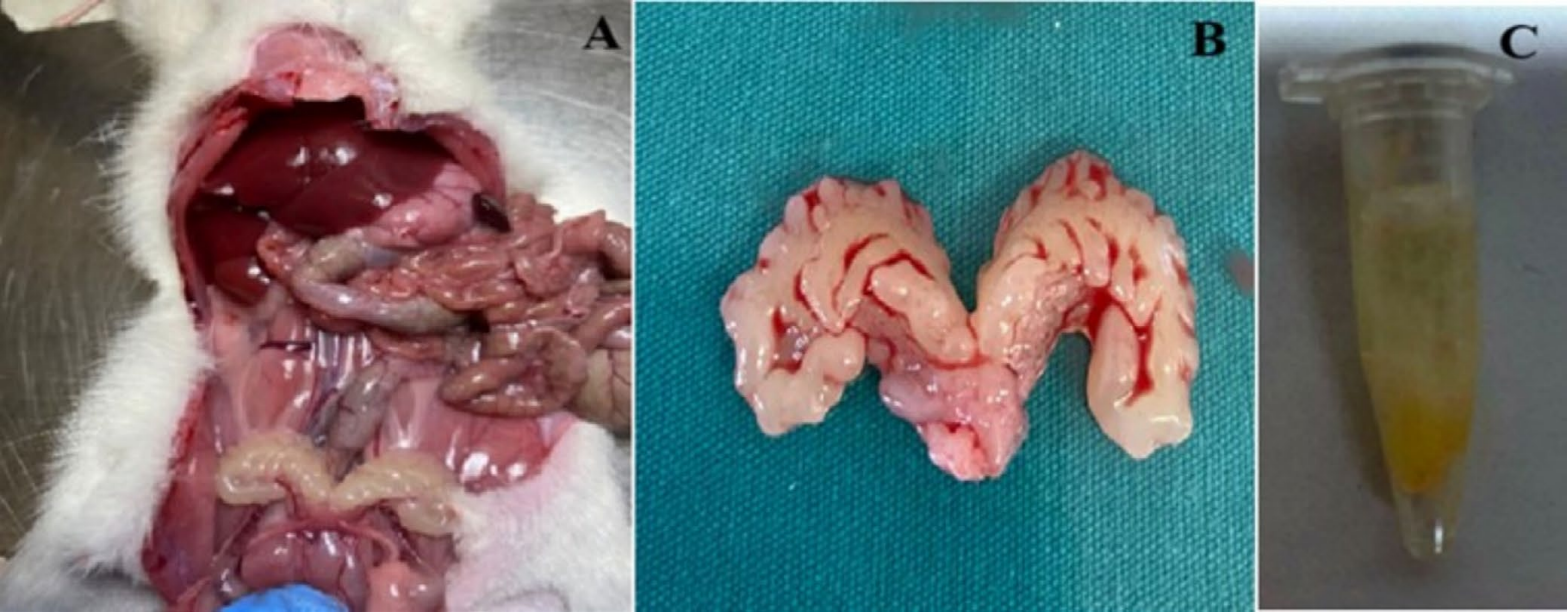
Seminal vesicle and sperm samples. (A, B) Seminal vesicle, (C) Sperm sample. The seminal vesicle was excised through intra‐abdominal laparotomy and subsequently placed in a petri dish for milking (A, B). The sperm samples collected were diluted with 1 cc of physiological serum (C) and subsequently transferred to tubes.

The seminal vesicle was excised to extract the contained sperm (Figure 2A,B). The sperm collected with a syringe (Figure 2C) was placed into tubes. The microscopic area underwent systematic scanning, with each sperm being evaluated. Motility was quantified by counting the total amount of immobile spermatozoa and expressed as a percentage. The motility value was categorized as either motile or immotile. The determination of sperm count was conducted utilizing a hemocytometer. From each well‐mixed sample, a 1:20 dilution ratio was achieved by combining 50 μL of epididymal spermatozoa in physiologically appropriate saline using 950 μL of diluent. The counts from both chambers of the hemocytometer were recorded, and the average was calculated, provided that the discrepancy between the two counts did not exceed 1/20 of the total, corresponding to a difference of less than 10%. Counts exceeding this threshold were discarded, requiring the sample dilution to be remixed and a new hemocytometer to be prepared. An average of six counts (three from each chamber) was then computed and expressed as 10^6^/mL (Ikebuaso et al. 2012).

### 2.4 Testosterone Analysis

Blood samples taken intracardially will be analyzed for testosterone using the ELISA method. The enzyme‐linked immunosorbent assay kit (BT‐lab catalog no. EA0023Ra) was analyzed using the reader for microplates. The BIO‐TEK EL X 800‐Auto strip washer was used, along with the BIO‐TEK EL X 50 device. 10 μL of the standard solution was utilized to allocate the specimens and regulate them into the coated wells. Following the addition of 100 μL of conjugate reagent and 50 μL of anti‐testosterone reagent, incubation was conducted for 90 min. The mixture was washed and incubated for an additional 20 min. The reaction will be terminated by adding 100 μL of 1 N hydrochloric acid. Absorbance was quantified at 450 nm with an automated spectrophotometer. The graph of absorbance against the concentration of the reference solution was plotted to obtain the standard curve, and the testosterone concentration was determined (Tietz 1995).

### 2.5 Calculation of Total Oxidant/Antioxidant Level and Oxidative Stress Index

TOS levels were quantified using commercially available kits (Rel assay, Turkey). The oxidants in the sample converted the ferrous ion‐o‐dianisidine combination to ferric ion. The presence of many glycerol molecules in the reaction media facilitated the oxidation reaction. The ferric ion formed a colorful combination with xylenol orange in an acidic environment. The color intensity, measured spectrophotometrically at 530 nm absorption (Mindray‐BS400), corresponded to the total oxidant molecule content in the sample. The assay was calibrated using hydrogen peroxide, and the results were expressed as μmol H_2_O_2_ equivalent/L (Erel 2005).

TAS was assessed utilizing commercially accessible kits (Rel assay, Turkey). The revolutionary automated technique depends on antioxidants decolorizing the distinctive hue of a more stable ABTS (2,2′‐Azino‐bis‐3‐ethylbenzothiazoline‐6‐sulfonic acid) radical cation. The experimental outcomes were analyzed using a spectrophotometer (Mindray‐BS400) set to an absorbance wavelength of 660 nm. The data were presented as mmol Trolox equivalent per liter (Erel 2004).

The ratio of TOS to TAS was defined as the OSI. The TAS unit was changed to μmol/L for calculation, and the OSI values were established using the following formula:

OSI (arbitrary unit) = TOS (μmol H_2_O_2_ equivalent/L)/TAC (μmol Trolox equivalent/L) (Harma et al. 2003; Kosecik et al. 2005; Yumru et al. 2009).

### 2.6 Volume Measurement

Testicular volumes were measured by the water displacement method. The volumes of the left testicular organs of each rat were calculated, and the mean value was obtained. It was considered a single observation and expressed in mL (Ikebuaso et al. 2012).

### 2.7 Histopathological Analysis

A segment of the biopsy samples from the testis was submerged in Bouin's reagent (Sigma‐Aldrich, St. Louis, MO, USA) at +4°C for 12 h, followed by dehydration via an ethanol classification series. The specimen was subsequently cleaned in xylene and fixed in paraffin. Serial cross‐sections, 5 μm in thickness, were obtained from paraffin‐embedded tissue blocks using a rotating microtome (Leica, Nussloch, Germany) and mounted on glass slides (Menzel Gläser, Braunschweig, Germany) for subsequent hematoxylin and eosin (HE) staining. The histopathological evaluation of the testicular tissue biopsy specimens was conducted using HE staining, in accordance with our previous research. The professionals assessed the HE‐stained slides using a bright‐field microscope (Olympus, Jena, Germany) to ascertain the histological characteristics and Johnsen scores (Johnsen 1970). A four‐tier grading system was introduced to assess histopathological alterations in testicular biopsies (Ozturk et al. 2016). Spermatogenesis was assessed by analyzing the cellular profile within the seminiferous tubules. Each sample underwent evaluation of seminiferous tubules, which were graded on a scale of 1–10 based on Johnsen's grading system (Johnsen 1970).

### 2.8 Statistical Analysis

The statistical analysis employed a general linear model‐ANCOVA to assess significant differences among the four groups: C, T/D, T/D + LD, and T/D + HD. This model was chosen because it satisfied the assumptions of parametric tests and facilitated the assessment of the collective impacts of Group, Testosterone, TAS, and TOS on the dependent variables, which include testicular volume, sperm count, and sperm motility. Spearman's rank correlation coefficient was utilized for correlation analysis, as it is suitable for non‐normally distributed data (evaluated using the Shapiro–Wilk test, *p* < 0.05). To mitigate the probability of Type I errors in multiple comparisons, the Bonferroni correction was employed where appropriate. Bioinformatics techniques and publicly accessible databases were employed for pathway enrichment analysis to find significantly enriched pathways linked to oxidative stress indicators. The criterion for statistical significance in pathway enrichment was established at *p* < 0.05, with modifications for multiple comparisons as required. For variables that failed to satisfy the assumptions of normality or homogeneity of variance, the Kruskal‐Wallis test was employed for multiple group comparisons, while the Mann–Whitney *U* test was utilized for pairwise comparisons. All statistical analyses were conducted utilizing IBM SPSS v22, with *p*‐values below 0.05 being statistically significant.

## 3 Results

### 3.1 Serum Sample Testosterone Hormone and Oxidative Stress Analysis

In 4 different variable parameters, control shows average, standard deviation, media, and average sorting values for T/D, T/D + LD, and T/D + HD. It indicates that there existed a substantial disparity between the groups for testosterone levels (*p* = 0.014). The average rank of the control group was the highest at 12.57, followed by the T/D + LD group at 22.94. The TAS levels among the groups were significantly different (*p* = 0.009). The control group showed the highest average ranking value, 23.29. Among all groups, the T/D + LD group exhibited the highest TAS value, second only to the control group. The TOS levels differed significantly among the groups (*p* = 0.021). The T/D had the highest average row with 22.38, while the T/D + LD had the lowest degrees with 8.50. OSI among the groups was with statistical significance (*p* = 0.004). The T/D reached the highest average of 24.25, while the T/D + LD had the lowest average row. This indicates that T/D has significantly superior performance compared to the T/D + LD group for general satisfaction, as shown in the high average ranks (Table 1).

**TABLE 1:** Intergroup values of serum testosterone levels and oxidative stress markers.

Variable	Group	Number	Mean	S.D.	Median	Mean rank	*p*
Testosterone	Control	7	4.24	1.15	3.89	12.57	0.014*
	T/D	8	3.82	1.67	3.25	9.38	
	T/D + LD	8	5.90	1.55	6.02	22.94	
	T/D + HD	8	4.69	1.04	4.36	18.69	
TAS	Control	7	1.81	0.23	1.87	23.29	0.009*
	T/D	8	1.41	0.33	1.34	9.75	
	T/D + LD	8	1.98	0.29	1.63	20.00	
	T/D + HD	8	1.41	0.07	1.41	11.88	
TOS	Control	7	3.36	1.86	2.42	18.00	0.021*
	T/D	8	4.58	2.09	5.01	22.38	
	T/D + LD	8	1.98	0.89	1.55	8.50	
	T/D + HD	8	2.75	1.07	2.61	15.38	
OS	Control	7	0.18	0.08	0.13	15.71	0.004*
	T/D	8	0.34	0.18	0.32	24.25	
	T/D + LD	8	0.12	0.06	0.11	7.63	
	T/D + HD	8	0.19	0.09	0.18	16.38	

*Note:* (1) Control (C), (2) Testicular torsion detorsion group (T/D), (3) Testicular torsion detorsion+Low dose hawthorn extract (T/D + LD), (4) Testicular torsion detorsion+High dose hawthorn extract (T/D + HD). Testosterone, Total Antioxidant Level (TAS), Total Oxidant Level (TOS), Oxidative Stress Index (OSI) parameters show statistical differences between the groups (*p* = 0.014, *p* = 0.009, *p* = 0.021, *p* = 0.004, respectively).

Abbreviation: SS, standard deviation.

* *p* < 0.05 is statistically significant. Kruskal‐Wallis H.

**FIGURE 3:**
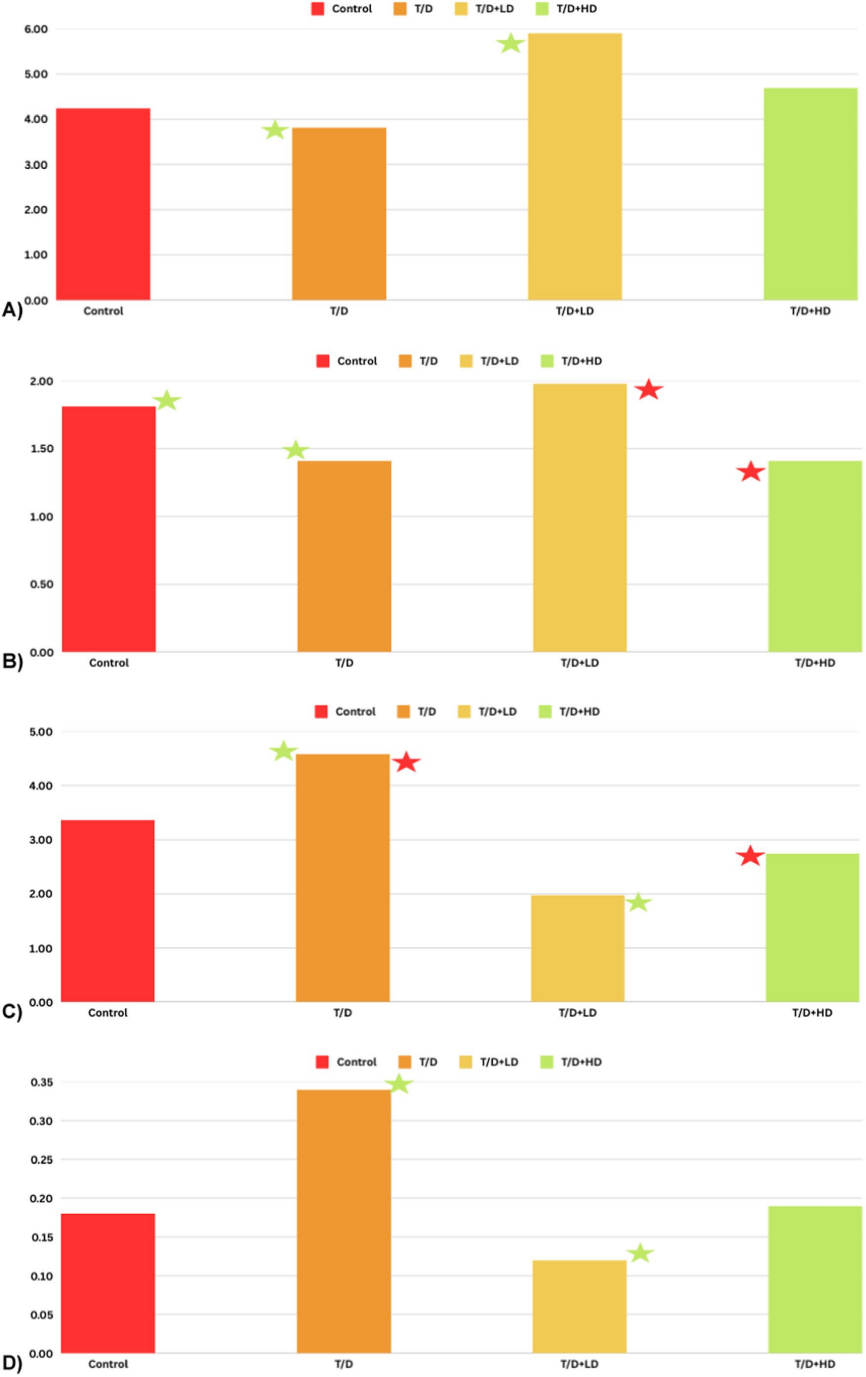
Comparison of testosterone and oxidative stress parameters between groups. (A) Testosterone level (ng/ml), (B) Total antioksidan Status (TAS mmol/L), (C) Total Oksidan Status (TOS μmol/L), (D) Oxidative Stress Index (OSI), 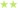 and 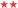 statistically significant. (1) Control (C), (2) Testicular torsion detorsion group (T/D), (3) Testicular torsion detorsion+Low dose hawthorn extract (T/D + LD), (4) Testicular torsion detorsion+High dose hawthorn extract (T/D + HD). In terms of testosterone level (ng/mL), T/D + LD and T/D + HD groups increased statistically significantly compared to the control group (*p* = 0.01, *p* = 0.008, respectively). In addition, it was observed that testosterone level increased statistically significantly compared to the T/D + HD control group (*p* = 0.008). In terms of TAS (mmol/L), TAS in T/D and T/D + HD groups increased statistically significantly compared to the control group (*p* = 0.02, *p* = 0.01, respectively). TAS in T/D + LD group increased statistically significantly compared to TD + HD (*p* = 0.01). TOS level in T/D group was statistically higher than T/D + LD and T/D + HD (*p* = 0.006, *p* = 0.04, respectively). In terms of OSI, T/D + LD OSI value decreased statistically significantly compared to control and T/D (*p* = 0.007, *p* = 0.001, respectively).

In terms of testosterone level (ng/ml), the T/D + LD and T/D + HD groups exhibited statistically significant differences relative to the control (both of them, *p* = 0.008). Furthermore, it was observed that testosterone levels increased statistically significantly in T/D + LD as opposed to T/D (*p* = 0.01). In terms of TAS (mmol/L), T/D, and T/D + HD decreased statistically significantly as opposed to the control (in order of *p* = 0.02, *p* = 0.01). TAS in T/D + LD increased statistically significantly compared to T/D + HD (*p* = 0.01). TOS in T/D was statistically elevated compared to T/D + LD and T/D + HD (*p* = 0.006, *p* = 0.04, respectively). TOS was higher than T/D + LD, statistically significant compared to control. In terms of OSI, T/D + LD OSI value decreased with statistical significance compared to the control and T/D (in order of *p* = 0.007, *p* = 0.001) (Figure 3).

The effect of the independent variables group, testosterone, TAS, and TOS, on testicular volume was analyzed (Table 1). The model explained 59.8% of the total variance (*R* ^2^ = 0.598, adjusted *R* ^2^ = 0.426). Testicular volume shows a significant effect on the dependent variable (*p* = 0.009) (Table 3). A significant difference was discovered between testicular volume as well as groups (*p* = 0.001, partial *η* ^2^ = 0.530) (Table 3). It was found that TOS had a significant effect on testicular volume (*p* = 0.033, partial *η* ^2^ = 0.199) (Table 3). A significant interaction was found between intergroup TOS levels (group * TOS) and testicular volume (*p* = 0.023, partial *η* ^2^ = 0.359) (Table 2).

**TABLE 2:** Parameters affecting testicular volume.

Dependent variable: Testis. volume. IDFN								
Source	Type III sum of squares	df	Mean square	*F*	Sig.	Partial eta squared	Noncent. Parameter	Observed power^b^
Corrected Model	0.341^a^	9	0.038	3.476	0.009	0.598	31.284	0.926
Intercept	0.249	1	0.249	22.812	0.000	0.521	22.812	0.995
Group	0.259	3	0.086	7.909	0.001*	0.530	23.726	0.972
Testosterone. IDFN	0.026	1	0.026	2.341	0.141	0.100	2341	0.309
TAS. IDFN	0.022	1	0.022	2.033	0.169	0.088	2033	0.275
TOS. IDFN	0.057	1	0.057	5.224	0.033*	0.199	5224	0.587
Group * TOS. IDFN	0.128	3	0.043	3.923	0.023*	0.359	110.770	0.750
Error	0.229	21	0.011					
Total	8.867	31						
Corrected Total	0.570	30						

*Note:* ANCOVA, adjusted *R* squared = 0.598 (adjusted *R* squared = 0.426), Total Antioxidant Level (TAS), Total Oxidant Level (TOS). Testicular volume shows statistically significant difference between groups (a, b) (*p* = 0.001). Testicular volume is affected statistically significantly by TOS and group (*p* = 0.033, *p* = 0.023, respectively).

* *p* < 0.05 is statistically significant.

Testosterone and TAS had no significant effect on testicular volume (in order of *p* = 0.141, *p* = 0.169), (Table 2). The control group had an adjusted mean value of Testicular Volume IDFN 0.659 (standard error: 0.044) and was greater than the other groups. The 95% confidence interval of the control was between 0.568 and 0.751, which was a potential indicator of a substantial disparity between the groupings. Testicular volume level in T/D + LD was statistically decreased as opposed to the control (*p* = 0.11). Testicular volume was statistically elevated in the T/D rather than the T/D + LD (*p* = 0.40). In addition, testicular volume was statistically decreased in the T/D + LD in comparison to T/D + HD (*p* = 0.14).

The distinction between the groups with regard to testicular volume was statistically significant (*p* = 0.019). Testicular volume level in T/D + LD was statistically decreased in comparison to the control (*p* = 0.11). Testicular volume was statistically elevated in the T/D in comparison to the T/D + LD (*p* = 0.40). In addition, testicular volume was statistically decreased in the T/D + LD in comparison to T/D + HD (*p* = 0.14) (Table 3).

**TABLE 3:** Comparison of testicular volume between groups.

Dependent Variable: Testis.volume. IDFN							
Group	Mean ± SD	Median	Mean rank	*p* *	*p* *	*p* *	*p* *
Control	0.60 ± 0.111	0.641	21.57	0.152^(1–2)^	0.011^(1–3)^ *	0.281^(1–4)^	0.019*
T/D	0.52 ± 0.090	0.534	15.88	0.152^(2–1)^	0.040^(2–3)^ *	0.878^(2–4)^	
T/D + LD	0.41 ± 0.064	0.403	7.14	0.011^(3–1)^ *	0.040^(3–2)^ *	0.014^(3–4)^ *	
T/D + HD	0.54 ± 0.099	0.494	17.13	0.281^(4–1)^	0.878^(4–2)^	0.014^(4–3)^ *	

*Note:* (1) Control (C); (2) Testicular torsion detorsion group (T/D); (3) Testicular torsion detorsion+Low dose hawthorn extract (T/D + LD); (4) Testicular torsion detorsion+High dose hawthorn extract (T/D + HD). Testicular volume level in T/D + LD was statistically decreased compared to the control group (*p* = 0.11). Testicular volume was statistically increased in the T/D group compared to the T/D + LD group (*p* = 0.40). In addition, testicular volume was statistically decreased in the T/D + LD group compared to T/D + HD (*p* = 0.14).

* *p* < 0.05 is statistically significant. Mann–Whitney *U* Kruskal‐Wallis H.

### 3.2 Sperm Cell Count and Morphological Analysis

**FIGURE 4:**
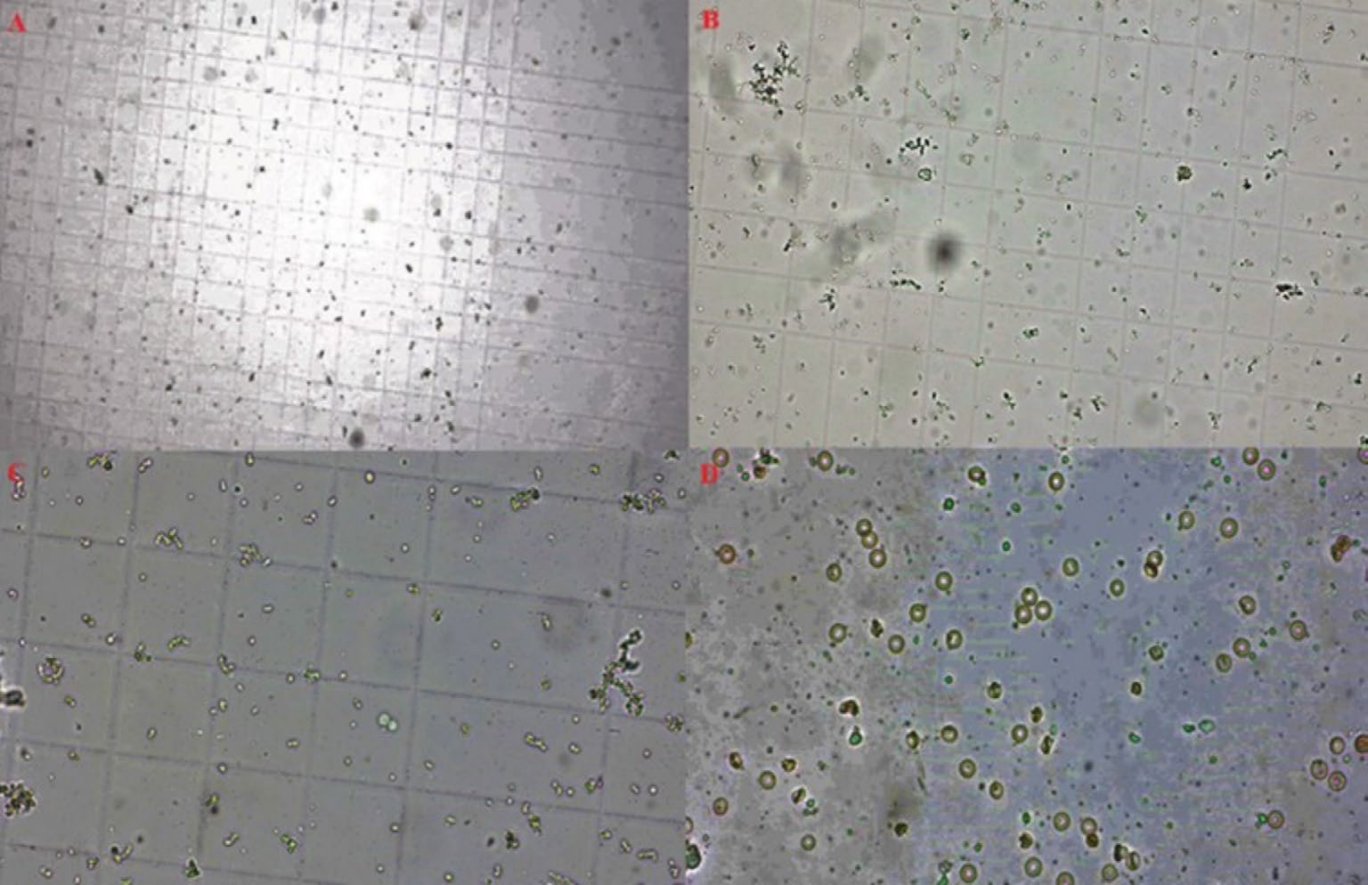
Sperm count and morphological assessment utilizing thoma slide. Sperm samples were positioned on a thoroughly cleaned and prepared Thoma slide. Samples were analyzed using fluorescence microscopy at varying magnification levels to assess sperm motility, morphology, and count. (A) Magnification of 10× was employed to evaluate the general area, (B) 20× magnification facilitated detailed observation of motility and sperm concentration, (C) 40× magnification allowed for a thorough analysis of the structural features (D) The application of 100× imaging facilitated a more precise evaluation of spermatogenesis, as well as sperm motility and quality. The volume calculation came after choosing a small square from each of the four sides and one from the center for the sperm count.

Sperm samples were placed on a pre‐cleaned and prepared Thoma slide. 100x imaging also allowed a more accurate assessment of the state of sperm motility and quality (Figure 4D). The data obtained were examined in the tables. Samples were examined under fluorescence microscopy at different magnification levels to assess sperm motility, morphology, and count. A 10× magnification was used to evaluate the general area (Figure 4A), a 20× magnification to observe motility in more detail and assess sperm concentration (Figure 4B), and a 40× magnification to analyze structural features (Figure 4C). Significant differences were detected among the groups based on sperm count (*p* = 0.000). The partial eta squared value (*η* ^2^ = 0.745) was 74.5% of the variance in sperm count of the groups, indicating that the effect of differences on sperm count was quite high. The number of sperm was statistically affected by only intragroup change (*p* = 0.00). Testosterone and TAS level did not significantly affect the number of sperm (Table 4).

**TABLE 4:** Parameters affecting sperm count and motility.

	Type III sum of squares	df	Mean square	*F*	Sig.	Partial eta squared	Noncent. parameter	Observed power^b^
*Sperm count*								
Corrected model	2990.443^a^	5	598.089	20.407	0.000	0.803	102.035	1.000
Intercept	734.209	1	734.209	25.052	0.000	0.501	25.052	0.998
Group	2143.701	3	714.567	24.381	0.000*	0.745	73.144	1.000
*Sperm motility*								
Corrected model	5336.100^a^	6	889.350	18.268	0.000	0.820	109.609	1.000
Intercept	2813.059	1	2813.059	57.783	0.000	0.707	57.783	1.000
Group	4684.174	3	1561.391	32.073	0.000*	0.800	96.218	1.000

*Note:* ANCOVA, sperm count adjusted R squared = 0.803 (adjusted R squared = 0.764), ANCOVA, sperm motility adjusted R squared = 0.820 (adjusted R squared = 0.775), *p* < 0.05 is statistically significant. Total Antioxidant Level (TAS). Sperm number and motility shows statistically significant difference between groups (a, b) (*p* = 0.00).

* *p* < 0.05 is statistically significant.

Sperm count of T/D was statistically significantly decreased compared to the control, T/D + LD, as well as T/D + HD (in order of *p* = 0.00, *p* = 0.021, *p* = 0.00). Sperm count of T/D + LD and T/D + HD groups was statistically significantly somewhat inferior to the control (in order of, *p* = 0.00, *p* = 0.01) (Table 5).

**TABLE 5:** Comparison of sperm count between groups.

(I) Group	(J) Group	Mean difference (I–J)	Std. error	Sig.^a^	95% confidence interval for difference^a^	
					Lower bound	Upper bound
Control	T/D	25.843*	3.222	0.000*	16.613	35.073
	T/D + LD	15.461*	2.902	0.000*	7.146	23.776
	T/D + HD	11.079*	3.159	0.010*	2.030	20.129
T/D	Control	−25.843*	3.222	0.000*	−35.073	−16.613
	T/D + LD	−10.382*	3.212	0.021*	−19.583	−1.181
	T/D + HD	−14.764*	2.934	0.000*	−23.171	−6.357
T/D + LD	Control	−15.461*	2.902	0.000*	−23.776	−7.146
	T/D	10.382*	3.212	0.021*	1.181	19.583
	T/D + HD	−4.382	2.905	0.864	−12.703	3.940
T/D + HD	Control	−11.079*	3.159	0.010*	−20.129	−2.030
	T/D	14.764*	2.934	0.000*	6.357	23.171
	T/D + LD	4.382	2.905	0.864	−3.940	12.703

*Note:* (1) Control (C); (2) Testicular torsion detorsion group (T/D); (3) Testicular torsion detorsion+Low dose hawthorn extract (T/D + LD); (4) Testicular torsion detorsion+High dose hawthorn extract (T/D + HD). Sperm count in the T/D group was statistically significantly decreased compared to the control, T/D + LD, and T/D + HD (*p* = 0.00, *p* = 0.021, *p* = 0.00, respectively). Sperm count in the T/D + LD and T/D + HD groups was statistically significantly lower than the control group (*p* = 0.00, *p* = 0.01, respectively). Based on estimated marginal means.

^a^ Adjustment for multiple comparisons: Bonferroni.

* The mean difference is significant at the, 0.05 level.

When independent variables affecting sperm count are analyzed, it is seen that the group variable has a significant effect on sperm motility by explaining 80% of the total variance (partial *η* ^2^ = 0.800). This reveals that different groups have a significant effect on sperm motility (*p* = 0.00) (Table 4). Testosterone, TAS, and TOS variables had no significant effect on sperm motility (*p* = 0.394, *p* = 0.398, *p* = 0.725) (Table 6). T/D group sperm motility decreased in comparison to T/D + HD and control (both of them *p* = 0.00). T/D + LD and T/D + HD sperm motility decreased in comparison to control (in order of *p* = 0.00, *p* = 0.001). T/D + HD sperm motility increased statistically in comparison to T/D (*p* = 0.00).

**TABLE 6:** Comparison of sperm motility between groups.

(I) Group	(J) Group	Mean difference (I–J)	Std. error	Sig.^a^	95% confidence interval for difference^a^	
					Lower bound	Upper bound
Control	T/D	37.296*	4.256	0.000*	25.061	49.531
	T/D + LD	26.140*	4.033	0.000*	14.545	37.735
	T/D + HD	18.546*	4.072	0.001*	6.839	30.252
T/D	Control	−37.296*	4.256	0.000*	−49.531	−25.061
	T/D + LD	−11.156	4.805	0.174	−24.970	2.657
	T/D + HD	−18.751*	3.907	0.000*	−29.985	−7.517
T/D + LD	Control	−26.140*	4.033	0.000*	−37.735	−14.545
	T/D	11.156	4.805	0.174	−2.657	24.970
	T/D + HD	−7.594	4.017	0.425	−19.145	3.956
T/D + HD	Control	−18.546*	4.072	0.001*	−30.252	−6.839
	T/D	18.751*	3.907	0.000*	7.517	29.985
	T/D + LD	7.594	4.017	0.425	−3.956	19.145

*Note:* (1) Control (C); (2) Testicular torsion detorsion group (T/D); (3) Testicular torsion detorsion+Low dose hawthorn extract (T/D + LD); (4) Testicular torsion detorsion+High dose hawthorn extract (T/D + HD). T/D group sperm motility decreased compared to T/D + HD and control (Both of them *p* = 0.00). T/D + LD and T/D + HD sperm motility decreased compared to control (*p* = 0.00, *p* = 0.001, respectively). T/D + HD sperm motility increased statistically compared to T/D (*p* = 0.00). Based on estimated marginal means.

^a^ Adjustment for multiple comparisons: Bonferroni.

* The mean difference is significant at the 0.05 level.

### 3.3 Testicular Tissue Histopathological Analysis

**FIGURE 5:**
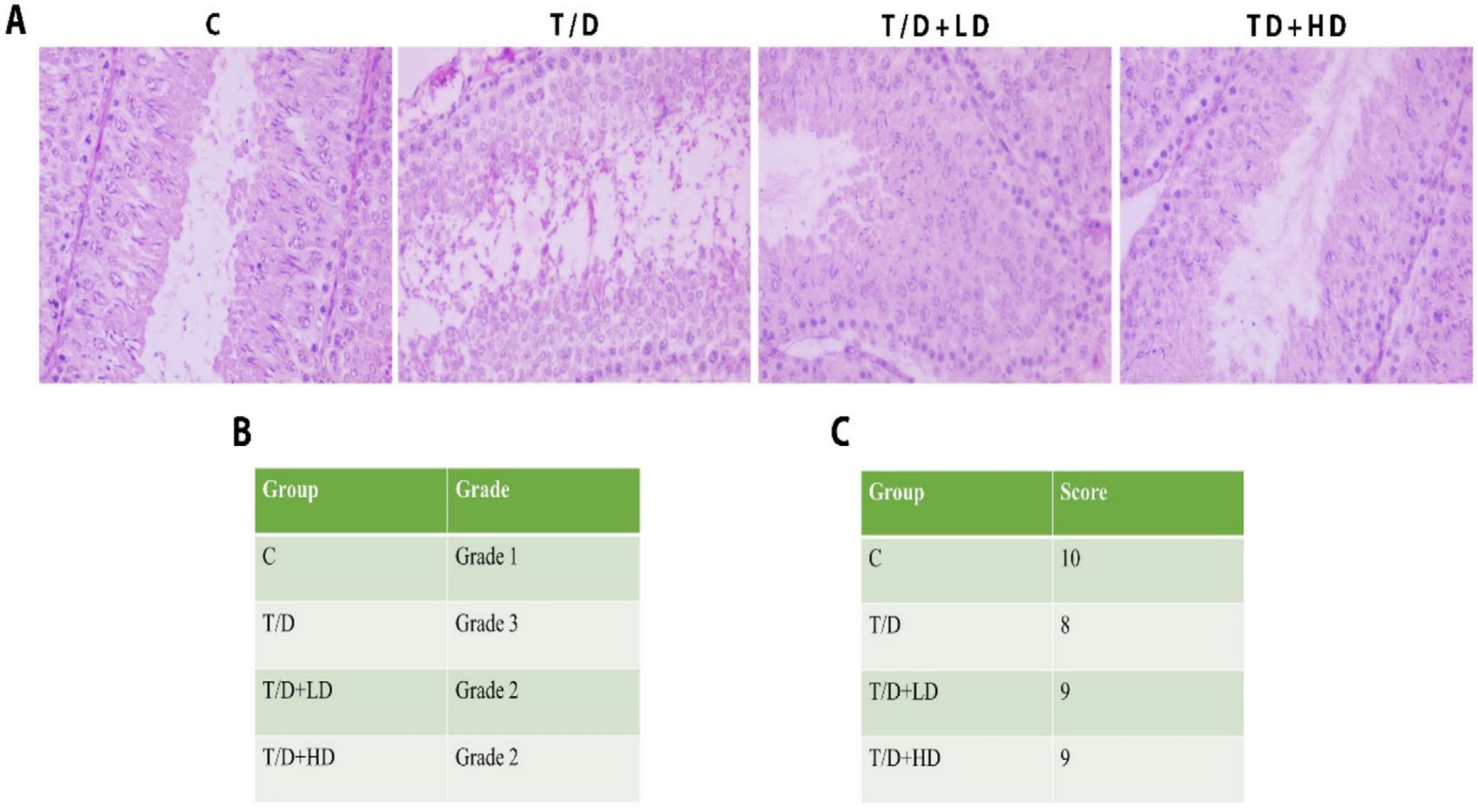
Histopathological evaluation of testis tissue. (A) Hematoxylin and eosin staining of groups, C, T/D, T/D + LD, T/D + HD, respectively. (B) Grade classification of the groups, (C) Johnsen scores of the groups. (1) Control (C), (2) Testicular torsion detorsion group (T/D), (3) Testicular torsion detorsion+Low dose hawthorn extract (T/D + LD), (4) Testicular torsion detorsion+High dose hawthorn extract (T/D + HD) (B, C); the hematoxylin and eosin‐stained testis sections were assessed using bright‐field microscopy to characterize their histopathologic features, Johnsen scores, and Cosentino's classification.

Testis sections stained with HE were assessed using bright‐field microscopes to describe the histopathologic features, Johnsen scores, and Cosentino's classification (Yesil et al. 2022) (Figure 5). The control group testes demonstrated complete spermatogenesis, with the presence of all germinal epithelial cells, including spermatogonia, primary and secondary spermatocytes, round, elongating, and elongated spermatids, spermatozoa, and Sertoli cells, as well as intertubular cells. The T/D group exhibited significant spermatogenic impairment, marked by a substantial decrease in spermatozoa and late spermatids. Both low‐ and high‐dose groups exhibited enhanced spermatogenesis, as evidenced by elevated late spermatid counts relative to the T/D group. The histopathological evaluation, Johnsen score, and Cosentino's classification results for all groups are presented in Figure 5.

## 4 Discussion

This study indicated that different amounts of hawthorn extract can help lower the damage that the testicular T/D model does to structure and function at the hormonal, oxidative, and histological levels. Significant differences in testosterone hormone, biochemical parameters (TAS, TOS, OSI), and histological evaluation (Grade and Johnsen classification) supported the protective effect of hawthorn extract on testicular tissue. Long‐term testicular torsion can cause testicular atrophy (Grimsby et al. 2018), testicular loss (Okorie 2011), germ cell necrosis (Shamsi‐Gamchi et al. 2018), spermatogenesis arrest (Turner et al. 2006), or lower serum testosterone (Turner et al. 2004). All of these things can make it impossible to get pregnant or have children. Testosterone is the androgen in the testis required to initiate and maintain spermatogenesis, and mature sperm production is tightly dependent on androgen action within the testis. Because of this, spermatogenesis stops at the meiosis stage when testosterone or its receptor is not present, which leads to male infertility (Grande et al. 2022). In our study, it was observed that testosterone hormone elevated significantly in the hawthorn extract groups in comparison to the T/D group (Table 1, Figure 3). The literature has demonstrated the significant effects of different flavonoids, including hawthorn extract, on testosterone levels in torsion models (Shokoohi et al. 2018; Soltani et al. 2018). According to our findings, the changes observed in TAS and TOS levels clearly show how the antioxidant properties of hawthorn extract can protect testicular tissue (Table 1, Figure 3). Lu et al. (2023) reported that hawthorn extract reduced oxidative stress and increased TAS levels, confirming its role in correcting intracellular oxidative imbalances (Lu et al. 2023). Gao et al. (2024) reported that hawthorn extract reduced oxidative stress and increased antioxidant defense in non‐alcoholic liver disease (Gao et al. 2024). Our study yielded similar results, showing that hawthorn extract suppressed oxidative stress through a reduction in TOS and an elevation in TAS (Table 1, Figure 3). These findings provide an important clue to understanding the biochemical basis of the way that hawthorn extract prevents testicular torsion. The potent antioxidant properties of flavonoids and proanthocyanidins may work by, among other things, scavenging free radicals and stopping lipid peroxidation (Parcheta et al. 2021). This also suggests a flavonoid effect of hawthorn extract.

The duration and extent of torsion in testicular torsion are of paramount significance. Timely diagnosis and surgical intervention for torsion are crucial for testicular preservation. Research indicates that if torsion is rectified within the initial 6 h, testicular salvage rates may attain 100%. Nonetheless, as the period of torsion extends, the probability of maintaining testicular viability diminishes progressively (Zvizdic et al. 2025). The extent of torsion directly influences testicular blood flow, hence elevating the risk of testicular necrosis.

Torsion over 360° markedly diminishes testicular blood flow, but torsion of 720° or above causes extended ischemia, culminating in irreparable testicular tissue destruction. According to the literature, at 1080° of torsion, 50% of testicles become irreparable (Herek et al. 2023). Consequently, both the duration and severity of testicular torsion are essential factors in therapeutic therapy. Our investigation employed a 720° torsion model consistent with existing literature (Soltani et al. 2018). This model was selected as it signifies a threshold where early detection and prompt intervention for testicular torsion are still viable. We did not directly quantify testicular blood flow; rather, we validated the stoppage of blood flow via macroscopic observation (Figure 1). This constraint hinders our capacity to directly correlate changes in blood flow with results from other studies in the literature.

Hawthorn extract exhibits significant antioxidant and anti‐inflammatory effects, potentially critical in alleviating ischemia–reperfusion injury linked to testicular torsion (Tadić et al. 2008; Yoo et al. 2016). Hawthorn extract contains flavonoids and proanthocyanidins that have been shown to scavenge free radicals, inhibit lipid peroxidation, and augment the activity of endogenous antioxidant enzymes, including superoxide dismutase (SOD) and glutathione peroxidase (GPx) (Shao et al. 2018; Tadić et al. 2008). Moreover, hawthorn extract has been shown to regulate cellular signaling pathways associated with oxidative stress and inflammation. Hawthorn extract has been demonstrated to activate the Nrf2/HO‐1 pathway, a principal regulator of the cellular antioxidant response (Yoo et al. 2016). This activation may result in the overexpression of cytoprotective genes and the augmentation of the cell's capacity to counteract oxidative damage. Moreover, hawthorn extract has been documented to demonstrate vasorelaxant and endothelium‐protective properties, potentially facilitated by enhanced nitric oxide (NO) synthesis and the modulation of endothelial nitric oxide synthase (eNOS) activity (Brixius et al. 2006; Leung and Mw Wong 2013). These vascular actions may enhance blood circulation and oxygen supply to the testicular tissue, hence alleviating the ischemic injury induced by testicular torsion.

Minas et al. (2023) demonstrated the effect of testosterone, TAS, and TOS parameters on spermatogenesis. To demonstrate this effect and to show whether hawthorn extract has a curative effect, we observed testicular volume, sperm count, and sperm motility before histologic data (Tables 2, 3, 4, 5, 6). We observed a significant decrease in testicular volume in the T/D in comparison to the control, and a significantly elevated level in testicular volume in the hawthorn‐treated group in comparison to the torsion group (Table 3). Consistent with the data we detected in our study, (Herek et al. 2023) ultrasonographically demonstrated that one of the clinical data points in the evaluation of testicular torsion is the short axis of the testicle, and the other is the testicular volume (Herek et al. 2023). Data suggest that fluctuations in testicular volume can significantly impact hormonal balance and overall reproductive health. Testosterone is essential for a variety of physiological functions, including maintenance of libido and muscle mass, and its interaction with antioxidant levels highlights the need for a comprehensive approach to men's health. This comprehensive analysis will advance our understanding of male reproductive physiology and facilitate the development of potential therapeutic approaches to optimize testicular function and hormonal balance (Table 2). Conversely, our findings indicate that testosterone hormone and TAS do not contribute to the decrease in testicular volume during torsion; instead, the level of TOS influences the testicular volume (Table 2). The fact that TOS level is high and leads to a decrease in testicular volume indicates that oxidative stress causes serious cellular damage in testicular tissue (Küçük et al. 2021; Şahin et al. 2024). This observation the importance of treatment approaches targeting oxidative stress, such as hawthorn extract, after testicular torsion. On the other hand, treatments specifically aimed at reducing oxidative stress, rather than testosterone or general antioxidant levels, may be more effective in protecting testicular tissue (Table 5). Sperm count also differed between the groups (Table 1), but was lower in the torsion group and elevated in the hawthorn extract treatment group (Figure 4, Tables 4 and 5). Only here we found that the higher dose of hawthorn was more significant (Tables 4 and 5), suggesting that hawthorn extract improves sperm quality. Sperm count alone is not a sufficient criterion to evaluate sperm quality. The scarcity and abundance of sperm have also been linked to infertility (Wang et al. 2021). Therefore, our study's evaluation of sperm motility revealed a decrease in sperm quality in the torsion model (Tables 5 and 6), while hawthorn extract emerged as a quality‐enhancing agent. Shahedi et al. (2021) showed that the effect on sperm motility was on antioxidant‐derived products (Shahedi et al. 2021). There are also studies showing that flavonoids increase sperm quality due to their antioxidant properties (Mishra et al. 2024). In order to study the mechanism of this effect, testosterone, TAS, and TOS were analyzed (Table 6). We found no significant correlation with all three parameters. This suggests that local cellular protection, membrane stabilization, improved energy metabolism, and support for testicular microvascular health, independent of testosterone, TAS, and TOS levels, likely mediate the positive effects of hawthorn extract on sperm motility and sperm count at different doses. It may also indicate that the antioxidant effects of hawthorn extract provide protection in testicular tissue not only through systemic parameters but also through specific mechanisms at the local cellular level.

In particular, following detorsion, the imbalance between ROS and the antioxidant defense system can lead to irreversible atrophy in testicular tissue. Additionally, the accumulation of ROS in the testes can result in a decrease in sperm concentration and viability, disruption of lipid composition, and increased DNA damage, all of which contribute to testicular damage. Over time, this becomes a significant factor leading to male infertility (Dokmeci et al. 2007; de Grezzana Filho et al. 2020; Küçük et al. 2021). Therefore, reducing oxidative stress parameters in testicular tissue after detorsion is of great importance. In recent years, studies on antioxidant agents aimed at preventing testicular atrophy and minimizing testicular damage have yielded promising results (Abadi et al. 2023; Moradi‐Ozarlou et al. 2020; Yuluğ et al. 2014). In this study, we aimed to demonstrate the antioxidant effect of hawthorn extract, showing that it may provide long‐term protective effects in detorsioned testicular tissue. Our findings, in line with the existing literature, suggest that hawthorn extract may have significant antioxidant properties, contributing to long‐term protection in detorsioned testicular tissue. These results provide a basis for future research and support the potential therapeutic application of hawthorn extract as a protective agent in testicular torsion treatment.

Histologically, higher scores in the Johnsen classification were observed in the groups treated with hawthorn extract in our study (Figure 5). For scores, the number of optimal tubules per tubule was observed; the tubules show that they work normally and that mature sperm production was optimal in the control group. In the hawthorn extract group, there were fewer sperm than in the control, but the number of sperm was higher in the tubule structure compared to the T/D (Figure 5). These suggest that hawthorn extract may have the ability to protect tubular structure and spermatogenesis. In our study, hawthorn extract was observed to support spermatogenesis and increase testicular tissue integrity by protecting both cellular and histologic structure in testicular tissue (Figure 5). These results indicate that hawthorn extract may be a potential therapeutic alternative against testicular T/D injury. Studies on herbal extracts, which evaluated testicular structure, tubule structure, and spermatogenesis, yielded positive results similar to our study (Nagy et al. 2024; Soltani et al. 2018). These findings reveal the potential of hawthorn extract to protect tubular structure and spermatogenesis by reducing the damage after testicular T/D and suggest that further investigation of the molecular mechanisms underlying the beneficial effects of herbal extracts on testicular tissue may open the door to new treatment approaches in male reproductive health.

This study makes important scientific contributions by comprehensively evaluating the protective effects of hawthorn extract against testicular torsion. The combined examination of both biochemical and histologic parameters increases the reliability and comprehensiveness of the findings. The measurement of a wide range of parameters, such as testosterone, TAS, TOS, OSI, and histological Johnsen scores, made it possible to analyze the effects of hawthorn extract from a multifaceted perspective. Furthermore, the randomization of the experimental groups and the availability of an appropriate control group reinforced the methodological soundness of the study and supported the accuracy of the results. This rigorous approach ensured that the data obtained was reproducible and reliable. The observation of protective effects of hawthorn extract on spermatogenesis and supportive effects on tissue integrity emphasizes the therapeutic potential of this herbal treatment option.

While significant findings were obtained, some limitations must be discussed for a clearer assessment of the results. The study was conducted on a limited number of animals, which may affect the statistical power and robustness of the findings. A larger sample size could provide more definitive conclusions and improve the reliability of statistical analyses. The study focused on the short‐term effects of hawthorn extract following testicular T/D. However, long‐term evaluations were not conducted, limiting the understanding of sustained protective effects and potential delayed adverse outcomes. While biochemical and histological assessments were performed, the study did not include molecular‐level investigations such as gene expression analysis, oxidative stress pathway assessments, or apoptotic markers. Incorporating these analyses could provide deeper mechanistic insights into the protective effects of hawthorn extract. Although the study included appropriate control groups, there was no standalone hawthorn extract group without ischemia–reperfusion injury. This could have provided additional insights into the direct effects of hawthorn extract on normal testicular physiology. To address these limitations, future studies should incorporate larger sample sizes to enhance statistical power, long‐term follow‐up studies to assess sustained protective effects, molecular‐level analyses to understand underlying mechanisms, additional control groups to isolate the effects of hawthorn extract, and human clinical trials to evaluate the extract's translational potential. Future research should aim to refine methodologies, expand analyses, and conduct translational studies to validate these findings in clinical settings. Recognizing these constraints will enhance the credibility and applicability of research in this field.

This study's findings establish a crucial foundation for assessing hawthorn extract's potential as a therapeutic agent to prevent testicular torsion‐induced damage. Our study will be a stepping stone for future studies to determine the effects of hawthorn extract in more detail. Large‐scale studies comparing the effects of different doses will be critical in determining the optimal doses of these herbal extracts. Furthermore, longer‐term experiments are necessary to understand the long‐term effects and lasting protective benefits of hawthorn extract. Thus, the long‐term safety and efficacy of hawthorn extract can be evaluated. Gene expression analysis and research into certain cellular signaling pathways are also needed to fully understand the molecular effects of hawthorn extract. These kinds of studies might help us understand more about how hawthorn extract affects spermatogenesis on a biological level. The findings could lead to the creation of new treatments for problems related to men's reproductive health. Therefore, this research is a stepping stone in understanding the therapeutic potential of hawthorn extract and transferring it to clinical applications.

## 5 Conclusion

This research indicated that hawthorn extract can protect against T/D injuries to the testicles and keep the tissue's integrity by lowering oxidative stress and encouraging spermatogenesis. Findings suggest that hawthorn extract may be a promising therapeutic agent in cases of acute testicular injury. However, to gain a deeper understanding of the long‐term effects and optimal dosage of hawthorn extract, large‐scale, long‐term studies investigating its mechanisms at the molecular level are needed. Such research will provide a more solid foundation for the potential use of hawthorn extract as a safe and effective treatment option in clinical practice.

## Author Contributions

**Ümmü Gülşen Bozok:** conceptualization (equal), formal analysis (equal), project administration (lead), resources (equal), validation (equal), visualization (equal), writing – original draft (equal), writing – review and editing (equal). **Gülbahar Böyük Özcan:** data curation (equal), formal analysis (equal), project administration (equal), visualization (equal). **Fatma Uysal Cinar:** visualization (equal).

## Ethics Statement

The ethical committee was approved by Ankara Medipol University Experimental Animals Ethics Committee with the number HDY2/2, dated 6/6/2024.

## Consent

The authors have nothing to report.

## Conflicts of Interest

The authors declare no conflicts of interest.

## Data Availability

The datasets used and/or analyzed during the current study are available from the corresponding author on reasonable request.
